# Update on Instrumentations for Cholecystectomies Performed via Transvaginal Route: State of the Art and Future Prospectives

**DOI:** 10.1155/2010/405469

**Published:** 2010-02-11

**Authors:** Elia Pulvirenti, Adriana Toro, Isidoro Di Carlo

**Affiliations:** Department of Surgical Sciences, Organ Transplantation, and Advanced Technologies, University of Catania, Cannizzaro Hospital, Via Messina 829, 95126 Catania, Italy

## Abstract

Natural Orifice Transluminal Endoscopic Surgery (NOTES) is an innovative approach in which a flexible endoscope enters the abdominal cavity via the transesophageal, transgastric, transcolonic, transvaginal or transvescical route, combining the technique of minimally invasive surgery with flexible endoscopy. Several groups have described different modifications by using flexible endoscopes with different levels of laparoscopic assistance. Transvaginal cholecystectomy (TVC) consists in accessing the abdominal cavity through a posterior colpotomy and using the vaginal incision as a visual or operative port. An increasing interest has arisen around the TVC; nevertheless, the most common and highlighted concern is about the lack of specific instruments dedicated to the vaginal access route. TVC should be distinguished between “pure”, in which the entire operation is performed through the transvaginal route, and “hybrid”, in which the colpotomy represents only a support to introduce instruments and the operation is performed mainly by the classic transabdominal-introduced instruments. Although this new technique seems very appealing for patients, on the other hand it is very challenging for the surgeon because of the difficulties related to the mode of access, the limited technology currently available and the risk of complications related to the organ utilized for access. In this brief review all the most recent advancements in the field of TVC's techniques and instrumentations are listed and discussed.

## 1. Introduction

Natural Orifice Transluminal Endoscopic Surgery (NOTES) is an innovative approach in which a flexible endoscope enters the abdominal cavity via the transesophageal, transgastric, transcolonic, transvaginal, or transvesical route, combining the technique of minimally invasive surgery with flexible endoscopy [1]. The first report of transvaginal cholecystectomy (TVC) is devised to the American gynecologist D. Tsin who firstly performed this revolutionary approach at Mount Sinai Hospital of New York in 2003 [2]. Since then, several groups have described different modifications by using flexible endoscopes with different levels of laparoscopic assistance. 

Although this new technique seems very appealing for patients, who prefer it to standard laparoscopy since it eliminates abdominal wall scares and reduces postoperative pain [3], it is very challenging for the surgeon because of the difficulties related to the mode of access, the limited technology currently available, and the risk of complications related to the organ utilized for access [4, 5]. Despite the huge interest in the development of NOTES and its applications in clinical practice, it still remains at present largely experimental [5].

In this brief review all the most recent advancements in the field of TVC's techniques and instrumentations are listed and discussed.

## 2. State of the Art

As described by the author who first performed it [2], TVC consists in accessing the abdominal cavity through a posterior colpotomy and using the vaginal incision as a visual or operative port. Pneumoperitoneum is achieved by introducing a 12 mm in diameter/15 mm in length trocar and, after insufflations are complete, the first trocar is replaced with a 10 mm scope which allows to introduce a 5 mm abdominal trocar under culdoscopic surveillance. According to this first report, the dissection of Calot's triangle and the cholecystectomy itself are performed by using the abdominal-inserted trocars. The vaginal route is used again only for the extraction of the specimen.

Since this initial description, an increasing interest has arisen around the TVC as demonstrated by the huge amount of articles published during the last five years, most of them including only small groups of patients [6, 7] or animal models. Nevertheless, the most common and highlighted concern is about the lack of specific instruments dedicated to the vaginal access route [1–8]. Current procedures with commercially available flexible endoscopes are technically limited by the inability to manipulate tissue or retract organs [9]. The flexibility of the endoscope shaft makes it difficult to exert sufficient distal force to manipulate the liver and to expose adequately the gallbladder: indeed, the endoscope can buckle against the hepatic lobe or move away, so that the classic traction/countertraction concept on which surgery is based is invalidated [9]. TVC shares many of the technical difficulties of laparoscopic surgery such as the use of long instruments through fixed angles but has additional problems related with orientation and rotation of the image [5]. For this reason TVC is actually slow and extremely demanding [5].

TVC should be distinguished into “pure”, in which the entire operation is performed through the transvaginal route, and “hybrid”, in which the colpotomy represents only a support to introduce instruments and the operation is performed mainly by the classic transabdominal-introduced instruments.

## 3. Pure TVC

Pure TVC avoids the need for laparoscopic assistance by introducing two flexible scopes into the abdominal cavity. De Sousa et al. [10] in 2008 described their experience of 4 women with symptomatic cholelitiasis. Due to the lack of available instruments, pneumoperitoneum is achieved by attaching a flexible plastic tube to a standard single-channel gastroscope (FUJINON, Japan) and by introducing it through a posterior 2.5 cm colpotomy performed under direct view. Subsequently, a second double-channel colonscope (FUJINON EC 410- D, FUJINON, Japan) is inserted through the same orifice. Gallbladder retraction is obtained by using the first endoscope, whilst dissection of Calot's triangle is performed through the second endoscope by using standard endoscopic instruments such us endoscopic hook, hot-biopsy forceps for cystic duct and artery dissection, polipectomy snare for gallbladder's dissection from the liver bed, and specimen extraction. According to the authors, spatial orientation and visualization were of good quality. The main difficulty was related to the fact that endoscopic devices have to be inserted into a working channel (i.e., the vagina), constraining the movement to the long axis of the endoscope, with a subsequent lack of triangulation.

Gumbs et al. [11] have recently described the first pure TVC performed in an American patient. Through a colpotomy achieved under direct visualization, a 15 mm port (Applied Medical, Rancho Santa Margarita, CA) is placed to obtain the pneumoperitoneum. Through this port, a double-channel gastroscope (Storz: 13806 NKS, Tuttlingen, Germany) is inserted and retroflexed to make sure that no pelvic or abdominal structures were injured after the first entry. The main difference comparing the previously mentioned experience is that the operative instrumentation was represented by an articulating extra long instrument (Novare, Cupertino, CA) placed into the abdomen through a second lateral colpotomy and not through the same incision. The endoscopic hook knife (Olympus Surgical America, Orangeburg, NY) and grasper biopsy forceps (Boston Scientific) are inserted in the channel of the gastroscope to dissect the structure of the Triangle of Calot. According to the experience of the authors, the most important barrier to the widespread adoption of pure TVC is the difficulty in obtaining the so-called “clinical view of safety”, specifically at the level of the Triangle of Calot, due to the fact that the endoscope comes posteriorly, with an increased risk of bile duct injury. The problem of achieving adequate tension on the tissues is addressed by performing a combination of grasping with the endoscopic grasper and cutting with the endoscopic cautery device. However, the most important limit encountered by the authors was the lack of commercially available clips to perform safely the closure of the cystic duct. For this reason, the tips of the available clips (Quickclips, Olympus Surgical America) were straightened manually by using a needle holder. Finally, it is important to emphasize how the desufflation results limited by the absence of the abdominal trocars, so that the pneumoperitoneum required the aspiration via the endoscope and, furthermore, the compression of the patient's chest and abdomen while leaving the transvaginal trocar open. In any case, a lower insufflation pressure is generally used because the endoscopes work at 3–5 cm of distance to the subject [10], so there is a reduced need of wide exposure.

As demonstrated by the small amount of patients treated up to now, pure TVC has still to be considered widely experimental and its application requires a multidisciplinary team and a logistic support that is difficult to obtain even in the most advanced centers. For this reason, and due to the lack of dedicated instruments, the great number of TVCs performed to date are still based on different levels of standard laparoscopic assistance. Further large series and randomized studies are required to evaluate the middle and long term results, especially with regards to the safety and the costs/benefits rate.

## 4. Hybrid TVC

Most authors share the opinion that it is not yet possible to perform TVCs without the help of instruments introduced through the abdominal wall [12]. For this reason the great amount of TVCs described in literature represents a fusion of laparoscopy and endoscopic surgery, with an approach defined as “hybrid”. 

In this field there is a remarkable lack of accordance about the difference between “laparoscopically assisted transvaginal surgery” and “transvaginally assisted laparoscopic surgery”. The first term indicates an operation performed mainly via the transvaginal access, in which most of the instruments assigned to perform the decisive dissection steps are inserted via this route and the abdominal access is used only as a support for retraction. Conversely, the second term refers to operations based mainly on the traditional laparoscopic approach and instrumentations, and the vaginal access allows the introduction of instruments for retraction. 

One of the largest series of “laparoscopically assisted transvaginal cholecystectomies” is reported by Horgan et al. [13]: in 5 patients a 5 mm umbilical trocar is inserted for abdominal exploration and to determine the feasibility of the vaginal access. This trocar represents the only trans-abdominally inserted instrument since all the subsequent operative steps are performed by instruments inserted through the vagina. The difficulties encountered with this approach are related to maintaining of pneumoperitoneum, since insufflation is more difficult to manage and measure than with a standard laparoscopic port, and to dissection of the gallbladder from the liver bed, which has resulted difficult by using the endoscopic device (Olympus America, Center Valley, PA, USA), as the small size makes dissection cumbersome and longer compared to classic laparoscopic approach. Eventually, the endoscopic clips that have been used are described as not entirely occlusive and not designate to secure the cystic duct safely, underlining the importance of focusing the research in order to overcome this limit.

In another work by Noguera et al. [12] a video endoscope is introduced through the colpotomy with a rigid 12 mm in diameter/15 mm in length trocar, with the advantages of maintaining pneumoperitoneum thanks to its retension valve, stabilizing the endoscope with its length, making the movements easier, and pointing the endoscope toward the gallbladder, so reducing the risk of bile ducts and liver injury. Through the working channel of the endoscope, instruments for grasping, dissecting, cutting, and sealing are introduced, so this approach can be defined as an “*intermediate* laparoscopically assisted transvaginal cholecystectomy”: indeed, despite the fact that two abdominal trocars are inserted, the main steps are performed through the vaginally inserted instruments.

On the contrary, a typical “transvaginally assisted laparoscopic cholecystectomy” is described by Ramos et al. [8] who combined their experience with bariatric and laparoscopic surgery in order to avoid the lack of specific instrumentation for TVC. In details, in 32 patients the transvaginal route was used only to introduce a 10 mm 45° rigid bariatric laparoscopic optic through a 12 mm bariatric trocar placed in the posterior vaginal fornix. The main steps of the operation were performed through a 5 mm intraumbilical port with classic laparoscopic instruments, but a 2 mm trocar had to be positioned on the right flank in order to retract the gallbladder. Also these authors have used the vagina to remove the specimen from the abdominal cavity. This work demonstrated that TVC performed by using only regular laparoscopic instruments and avoiding the introduction of endoscopes can be safely performed until specific endoscopic instruments are under development, also minimizing the need to acquire new technical skills.

A similar experience is reported by Pugliese et al. [14] who performed TVC in 18 women by introducing a 45 cm long grasper and a double-channel Karl Storz flexible gastroscope (Tuttlingen, Germany) through the colpotomy. The endoscope represents the only source of light during the procedure, whilst the grasper is used to achieve traction of the gallbladder, but not dissection. All the procedures were accomplished by using a single trocar of 5 mm placed in the left hypochondrium and not in the umbilical area to obtain a more ergonomic operative position to manipulate the gallbladder in combination with the transvaginal grasper, especially to fire clips. In contrast with authors who prefer standard rigid devices [8], these authors prefer using flexible endoscope to reach the supramesocolic region, so that the view of the biliary structures is facilitated by the flexibility of the endoscopic tips. However, endoscopes are not considered useful and safe for dissection, so this step is performed through the abdominal trocar, using, if necessary, the transvaginally inserted grasper only as a support for dissection in case of difficulties. This approach overcomes two current limits of NOTES available instruments: first, they are not flexible, long, or thin enough to reach good angulation for dissection; and second, the working channel of flexible endoscopes is too close and parallel, hindering an effective manipulation [14].

Analogously to what was previously said, an “*intermediate *transvaginally assisted laparoscopic cholecystectomy” approach is performed by Zornig et al. [1] who described one of the largest series reported in literature in which the decisive steps are performed through the umbilical port, but two instruments are inserted in vagina and only one in the umbilicus. In details, after the induction of pneumoperitoneum with a Verres needle and after performing the exploration of the abdominal cavity with a 5 mm optic, a 5 mm mandrin is inserted in the posterior fornix of the vagina and replaced by a 5 mm extra long dissector (Storz, Tuttlingen, Germany), with a 10 mm trocar inserted alongside. Through this trocar, an extra long 10 mm optic (Olympus, Hamburg, Germany) is inserted, and the umbilical optic is replaced with another dissector. Cystic duct and cystic artery dissection, positioning of clips, and gallbladder's mobilization are performed via the umbilicus by traditional laparoscopic instruments. The 10 mm vaginal trocar is used again only for specimen's extraction inside a removal bag. This large series has demonstrated that hybrid TVC is feasible in adult women of any age, even obese, previously operated or with gallbladder phlogosis. However, these authors experienced a difficulty related to the fact that the traditional flexible instruments available today for endoscopic procedures are much more difficult to use for abdominal surgery, resulting in a longer time requiring for performing the whole operation.

## 5. Future Prospectives

Endoscopes for TVC are required to have high resolution, large operative channels, some degree of triangulation, and a length suitable to reach any intraabdominal point. Today, only a small amount of devices address most of these points [15]. In particular, the “R” scope from Olympus and the Transport scope from USGI medical may address some problems related to access and visualization, whilst the Eagle Claw (Olympus), the Swain system (Ethicon), and the G-prox (USGI) seem able to secure better intraoperative performances, but they are still under development [15].

Many disadvantages still limit the chances of early routine operations (i.e., unstable vision, early state of development, etc.) [16]. However, the medical devices companies are racing to solve these problems and the industry is quickly producing futuristically designed instruments to overcome the barriers in TVC progress [17, 18].

Better cosmetic results, fewer wound infection rates, fewer trocar hernias, reduction or abolition of pain, and shorter hospital stay, although still theoretical, represent the strongest motivations in order to achieve the development in technology that this emerging field requires.

## References

[1] Zornig C, Mofid H, Siemssen L (2009). Transvaginal NOTES hybrid cholecystectomy: feasibility results in 68 cases with mid-term follow-up. *Endoscopy*.

[2] Tsin DA, Sequeria RJ, Giannikas G (2003). Culdolaparoscopic cholecystectomy during vaginal hysterectomy. *Journal of the Society of Laparoendoscopic Surgeons*.

[3] Varadarajulu S, Tamhane A, Drelichman ER (2008). Patient perception of natural orifice transluminal endoscopic surgery as a technique for cholecystectomy. *Gastrointestinal Endoscopy*.

[4] Forgione A (2009). In vivo microrobots for natural orifice transluminal surgery. Current status and future perspectives. *Surgical Oncology*.

[5] O’Riordain MG (2009). Cautionary considerations regarding N.O.T.E.S. in oncology. *Surgical Oncology*.

[6] Marescaux J, Dallemagne B, Perretta S, Wattiez A, Mutter D, Coumaros D (2007). Surgery without scars: report of transluminal cholecystectomy in a human being. *Archives of Surgery*.

[7] Zorrón R, Filgueiras M, Maggioni LC, Pombo L, Lopes Carvalho G, Lacerda Oliveira A (2007). NOTES transvaginal cholecystectomy: report of the first case. *Surgical Innovation*.

[8] Ramos AC, Murakami AHF, Galvão Neto M (2008). NOTES transvaginal video-assisted cholecystectomy: first series. *Endoscopy*.

[9] Ryou M, Thompson CC (2009). Magnetic retraction in natural-orifice transluminal endoscopic surgery (NOTES): addressing the problem of traction and countertraction. *Endoscopy*.

[10] de Sousa LH, de Sousa JAG, de Sousa Filho LH Totally NOTES (T-NOTES) transvaginal cholecystectomy using two endoscopes: preliminary report.

[11] Gumbs AA, Fowler D, Milone L (2009). Transvaginal natural orifice translumenal endoscopic surgery cholecystectomy: early evolution of the technique. *Annals of Surgery*.

[12] Noguera J, Dolz C, Cuadrado A, Olea J, Vilella A, Morales R (2009). Hybrid transvaginal cholecystectomy, NOTES, and minilaparoscopy: analysis of a prospective clinical series. *Surgical Endoscopy*.

[13] Horgan S, Cullen JP, Talamini MA (2009). Natural orifice surgery: initial clinical experience. *Surgical Endoscopy*.

[14] Pugliese R, Forgione A, Sansonna F, Ferrari GC, Di Lernia S, Magistro C Hybrid NOTES transvaginal cholecystectomy: operative and long-term results after 18 cases.

[15] Swanström LL (2009). Natural orifice transluminal endoscopic surgery. *Endoscopy*.

[16] Buess G, Becerra-Garcia F, Misra MC (2008). Instruments for transluminal laparoscopic surgery or “NOTES”. *Minimally Invasive Therapy and Allied Technologies*.

[17] Al-Akash M, Boyle E, Tanner WA (2009). N.O.T.E.S.: the progression of a novel and emerging technique. *Surgical Oncology*.

[18] Swanström L, Swain P, Denk P (2009). Development and validation of a new generation of flexible endoscope for NOTES. *Surgical Innovation*.

